# Antimicrobial Resistance in *Enterobacterales* Bacilli Isolated from Bloodstream Infection in Surgical Patients of Polish Hospitals

**DOI:** 10.1155/2021/6687148

**Published:** 2021-01-16

**Authors:** M. Kłos, M. Pomorska-Wesołowska, D. Romaniszyn, J. Wójkowska-Mach, A. Chmielarczyk

**Affiliations:** ^1^Faculty of Health Sciences, Jagiellonian University Medical College, Kraków, Poland; ^2^Department of Microbiology, Analytical and Microbiological Laboratory, KORLAB, Ruda Slaska, Poland; ^3^Department of Microbiology, Faculty of Medicine, Jagiellonian University Medical College, Kraków, Poland

## Abstract

**Background and Aims:**

Bloodstream infections (BSIs) are one of the most frequently observed hospital-acquired infections (HAIs). We sought to describe the epidemiology and drug resistance secondary *Enterobacterales* BSIs in surgical patients and check for any correlation with the type of hospital ward.

**Materials and Methods:**

This multicenter (13 hospitals in southern Poland) laboratory-based retrospective study evaluated adults diagnosed with BSI secondary to surgical site infection (SSI) hospitalized in 2015–2018; 121 *Enterobacterales* strains were collected. The drug resistance was tested according to the EUCAST recommendations. Tests confirming the presence of extended-spectrum *β*-lactamases (ESBLs) and *bla* resistance genes were carried out. The occurrence of possible clonal epidemics among *K. pneumoniae* strains was examined.

**Results:**

The prevalence of *Enterobacterales* in secondary BSI was 12.1%; the most common strains were *E. coli* (*n* = 74, 61.2%) and *Klebsiella* spp. (*n* = 33, 27.2%). High resistance involved ampicillin and ampicillin/sulbactam (92, 8–100%), fluoroquinolones (48–73%), and most cephalosporins (29–50%). Carbapenems were the antimicrobials with the susceptibility at 98%. The prevalence of ESBL strains was 37.2% (*n* = 45). All the ESBL strains had *bla*_CTX-M_ gene, 26.7% had the *bla*_SHV_ gene, and 24.4% had *bla*_TEM_ gene. The diversity of *Klebsiella* strains was relatively high. Only 4 strains belonged to one clone.

**Conclusions:**

What is particularly worrying is the high prevalence of *Enterobacterales* in BSI, as well as the high resistance to antimicrobial agents often used in the empirical therapy. To improve the effectiveness of empirical treatment in surgical departments, we need to know the epidemiology of both surgical site infection and BSI, secondary to SSI. We were surprised to note high heterogeneity among *K. pneumoniae* strains, which was different from our previous experience.

## 1. Introduction

In recent years, data concerning the epidemiology of bloodstream infections (BSI) have indicated a change in the prevalence of their microbial etiological agents. Gram-negative pathogens are predominant as the major causes of bacteremia, and according to researchers, the proportion of *Escherichia coli* increased by 33% and amounted to as many as ¼ of all pathogens and among patients of over 75 years of age, even by over 30% [1]. *Enterobacterales* bloodstream infection (BSI) is one of the most frequently observed hospital-acquired infections (HAIs), especially in surgical patients. All over the world, also in Poland, there are significantly more secondary BSIs, a fact that calls for immediate measures taken in order to prevent and control BSI in adults.

In 1983, strains with the extended-spectrum *β*-lactamase (ESBL) mechanism were first observed, and this phenotype quickly became dominant among Gram-negative resistant strains. Genes transmitted mainly by plasmids are responsible for resistance to penicillins, cephalosporins, and aztreonam, and their presence is also associated with resistance to antibiotics other than *β*-lactams, i.e., fluoroquinolones or aminoglycosides [2]. For more than twenty years, an increase in the number of resistant strains and infections with ESBL strains has been observed [2]. Widespread use of inexpensive antibiotics such as cephalosporins leads to the spread of ESBL strains in Europe and the USA [3]. Among the ESBL strains, *E. coli* and *Klebsiella pneumoniae* most often cause BSI [2, 4].

Studies by Sakellariou et al. have shown that *E.coli* BSI is most often of urological origin, whereas *K. pneumoniae* BSI is associated with surgical site infection (SSI), respiratory tract infection, or unknown infection [5]. Factors increasing the risk of such infections include the presence of invasive devices, especially vascular or indwelling catheters, age, ICU stay, septic shock, stay in a nursing home, and previous antibiotic therapy or inappropriate therapy [4, 6]. Antibiotic therapy with third-generation cephalosporins, *β*-lactams, and fluoroquinolones is particularly significant in the development of the ESBL phenotype [4]. ESBL infections are associated with increased mortality, costs, and prolonged hospitalization [2]. In the case of ESBL-positive *K. pneumoniae*, associated with surgical site infection, the risk factors include peritonitis, the use of broad-spectrum cephalosporins, neutropenia, septic shock, and ICU stay [7].

The main purpose of this study was to determine the drug resistance in *Enterobacterales* isolated from secondary bloodstream infection in surgical patients. In this manuscript, only cases of laboratory-confirmed bloodstream infections (LC-BSIs) were subjected to analysis according to the ECDC (European Centre for Disease Prevention and Control) definitions, in which it is diagnosed when the patient demonstrates at least one of the following symptoms: fever (>38°C), chills, blood pressure drop, blood cultures giving at least one positive result and a pathogenic microorganism is isolated, or two independent trials, up to 48 hours apart, give rise [8]. Additionally, the occurrence of possible clonal epidemics among *K. pneumoniae* strains was examined.

## 2. Materials and Methods

This multicenter laboratory-based retrospective study included 997 adult surgery patients (18≤) with LC-BSIs hospitalized in southern Poland (13 hospitals). The patients were hospitalized in 15 surgery units of different types: general, urology and vascular surgery wards, or gynecology and obstetrics wards. The qualifying criterion was to diagnose secondary bloodstream infection, except for central or peripheral venous catheter BSI.

Altogether, 121 *Enterobacterales* strains were isolated. Identification of microorganisms was performed using the MALDI-TOF Biotyper (Bruker Corporation, the Netherlands) according to standard methods. The isolates had been collected retrospectively in collaboration with KORLAB between 1 January 2015 and 31 December 2018 and the Department of Microbiology at the Jagiellonian University Medical College.

Antimicrobial susceptibility testing of all isolates was performed according to the current guidelines of the European Committee on Antimicrobial Susceptibility Testing (EUCAST, http://www.eucast.org/clinical_breakpoints/; accessed December 2017) by disc diffusion on Müeller–Hinton agar plates. Resistance was determined to six categories of antimicrobial agents: penicillin (also with clavulanic acid and tazobactam), cephalosporins (fourth-generation cefuroxime, ceftazidime, cefotaxime, cefepime, and cefoperazone-sulbactam), carbapenems (ertapenem, imipenem, and meropenem), aminoglycosides (amikacin, gentamicin, and tobramycin), fluoroquinolones (ciprofloxacin), and sulfamethoxazole with trimethoprim.

ESBL activity was detected with a modified double disk synergy test using a combination of cefotaxime, ceftazidime, cefepime, and aztreonam discs, placed 20 mm apart around a disc containing amoxicillin/clavulanic acid [9].

All isolates with ESBL activity were screened with multiplex PCR for the presence of *bla*_TEM_, *bla*_SHV_, and *bla*_OXA_*β*-lactamase genes and for variants of *bla*_CTX-M_*β*-lactamase genes (group including *bla*_CTX-M-1_, *bla*_CTX-M-3_, and *bla*_CTX-M-15_, group 2 including *bla*_CTX-M-2_, and group 9 including *bla*_CTX-M-9_ and *bla*_CTX-M-14_) using previously published primers [10]. Due to the largest number of ESBLs among *Klebsiella* spp. being *Klebsiella pneumoniae* strains (*n* = 27), pulsed-field gel electrophoresis (PFGE) was performed to check for a clonal epidemic. Our previous experiments showed that *Klebsiella* strains isolated in Polish departments often belong to one clone. PFGE was used to determine the possible horizontal transfer of *K. pneumoniae* strains among patients from the same wards. Genomic DNA was digested with 10 U *Xba*I (EURx, Gdańsk, Poland). The resulting DNA fingerprinting was obtained using the CHEF III PFGE system (BioRad, Warsaw, Poland) in 0.5 Tris-borate-EDTA buffer at 14°C at 6 V for 22 h with a ramped pulse time of 2–35 s. The GelCompar (Applied Maths) was used for cluster analysis using the Dice coefficient and the unweighted pair group method with arithmetic mean.

## 3. Results

The median age (quartiles Q1 and Q3) was 67 years (59, 76), and 63 (52.0%) patients were women. Most patients (47 persons, 38.8%) were hospitalized in general surgery wards. In the range of less than 59 years, a higher number of cases among women were observed compared to men (39.6% and 13.7%, respectively); while with age, there is an increase in the number of cases among men (in the range of over 70 years: men 51.7%, women 26.9%).

The prevalence of *Enterobacterales* in secondary BSI was 12.1%; the most common strain was *E. coli* (*n* = 74, 61.2%) and *Klebsiella* spp. (33 patients, 27.2%), regardless of the gender or age group (Table 1). In the genus *Klebsiella*, 27 *Klebsiella pneumoniae*, 5 *Klebsiella oxytoca*, and 1 *Klebsiella mobilis* strains were identified. An inconsiderable percentage of other species of *Enterobacterales* bacilli was 11.5%. In the group designated “others,” 10 strains were *Proteus mirabilis*, 3 *Enterobacter cloacae*, and 1 *Citrobacter freundii*.

The highest drug resistance was reported with regard to ampicillin in association with sulbactam, reaching 100% (Table 2). High resistance also involved fluoroquinolones (48–73%) and most cephalosporins (29–50%). Only 1 (0.8%) strain was fully sensitive, but 37 strains (30.6%) were resistant to 5 or more antimicrobial categories studied (Table 2). The studied strains demonstrated almost full sensitivity only to carbapenems (nearly 100% sensitivity), and two strains (*E. coli* and *Klebsiella pneumoniae*) were observed to be resistant to selected carbapenems.

The prevalence of ESBL strains was 37.2% (*n* = 45). Most ESBL-positive strains belonged to the genus *Klebsiella* (*n* = 22; 66.7%) and were especially present in general surgery wards (*n* = 175; 1.5%). Among *E. coli*, although they constitute the majority of the *Enterobacterales* strains isolated, ESBL strains were about ¼ (Table 3).

Among the ESBL (*n* = 45) strains, 12 strains (26.7%) had the *bla*_SHV_ gene (10 *Klebsiella* and 2 others), 11 strains (24.4%) had *bla*_TEM_ (6 *Klebsiella*, 3 others, and 2 *E. coli*), 14 strains (31.1%) had *bla*_OXA_ (6 *E. coli*, 6 *Klebsiella*, and 2 others), and 45 strains (100%) had *bla*_CTX-M_ (Table 3).

The most common were *bla*_CTX-M_ genes belonging to group 1 (including *bla*_CTX-M-1_, *bla*_CTX-M-3_, and *bla*_CTX-M-15_) up to 34 strains (75.5%), then variants of *bla*_CTX-M_ group 9 (including *bla*_CTX-M-9_ and *bla*_CTX-M-14_) in 4 strains (8.9%), and group 2 (including *bla*_CTX-M-2_) in one strain (2.2%). Seven of the *bla*_CTX-M_ genes belonged to groups other than those tested.

**Figure 1:**
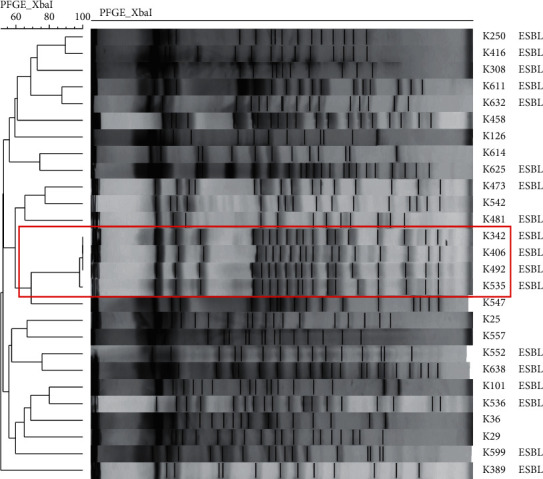
Pulsed-field gel electrophoresis dendrogram of *Klebsiella pneumoniae* strains.

The result of PFGE, among the studied *Klebsiella pneumoniae* strains, showed that only 4 strains that have been derived from the same ward of general surgery belonged to one clone. Other strains varied widely (Figure 1).

## 4. Discussion

*Enterobacterales* infections are common in surgical wards, but the presented data show that their prevalence depends significantly on the type of surgery procedures. In the analyzed population, they were most often identified in general surgery and after urological procedures, while in other populations, this type of etiology was rare.

According to Kolpa et al., the prevalence of *Enterobacteriaceae* in HAIs in patients after neurosurgical procedures was 25.8%, but mainly related to BSI cases, where prevalence was as high as 29.8% [11]. Similar values (33%) were obtained in pediatric cardiac surgery [12]. And as expected, completely different results were obtained in studies on infections in patients after urological procedures, where *Enterobacteriaceae* in BSI constituted 67.9%. The strains from LC-BSI showed significant drug resistance. They were highly susceptible only to ciprofloxacin, piperacillin-tazobactam (90%), meropenem, and gentamicin (100%) [13].

According to Melzer et al., urinary catheters increase the risk of severe bacteremia and should be used only when there is a clinical indication [14]. Research results by Lillie et al. confirm that one of the main reasons for the increase in BSI is the use of urinary catheters, often unnecessary [15]. Probably, the best way to reduce the number of BSI among urological patients is to reduce the indwelling catheters utilization and shortening the urinary catheter use [16].

To improve the effectiveness of empirical treatment in surgical departments, we need to know the epidemiology of both surgical site infection and BSI secondary to SSI, including the prevalence of their microbial etiological agents and antibiotic resistance profiles. Our results indicate an alarmingly high level of drug resistance in *Enterobacterales* strains associated with secondary BSIs in surgery patients in southern Poland. This situation may affect the risk of therapeutic failures in empirical therapy. Based on presented data in studied population, effective empirical therapy is possible only with cefoperazone-sulbactam or piperacillin-tazobactam in combination with aminoglycosides or the use of carbapenems, which is definitely unfavorable because it carries a high risk of increasing resistance to carbapenems among *Enterobacterales* which is already a big problem in other European countries such as Romania, Greece, and Italy [17]. In Poland, among *E. coli* and *K. pneumoniae* strains, resistance to aminopenicillins, third-generation cephalosporins, and fluoroquinolones is still a bigger problem, which does not mean that we should not be afraid of the appearance of carbapenem-resistant strains [18]. Fortunately, resistance to carbapenems in *Enterobacterales* was not confirmed in our studies, as only one strain was resistant to imipenem. However, the application of one of the antibiotics from the group of *β*-lactams, carbapenems, aminoglycosides, or fluoroquinolones may lead to coselection resistance to several groups of antibiotics. This is an indication for the need to perform microbiological tests concurrently with targeted therapy and to conduct precise microbiological surveillance. Unfortunately, in Polish hospitals, microbiological testing is underutilized and antibiotic consumption is one of the highest in Europe [19]. On the other hand, measures are required to implement appropriate infection prevention and control procedures.

Despite the fact that no mechanisms of carbapenem resistance have been found in our study, we still observe a high level of ESBL-positive strains, especially in *K. pneumoniae*. This observation is consistent with reports from the SENTRY Antimicrobial Surveillance Program, which indicate a continuous and faster increase in the share of ESBL-positive *K. pneumoniae* and *E. coli* strains in various types of infections in hospitals in the US [20]. In our 2011–2013 research, the presence of ESBL was only slightly lower at 57% KP and 21.4% EC [21]. In addition, ESBL strains isolated from newborns were resistant to aminoglycosides and SXT as here. Molecular characterization of the 45 ESBL-producing isolates showed that all (100%) isolates were producing *bla*_CTX-M_-type ESBLs, which represented most of all by groups *bla*_CTX-M-1_, *bla*_CTX-M-3_, and *bla*_CTX-M-15_ (75.5%), which is consistent with our other studies about ESBL strains in southern Poland [21, 22].

Molecular studies suggest that both mechanisms (clonal spread and plasmid transfer) are important since ESBL-positive strains have a larger share of the outbreak than others. The abundance of ESBL-positive strains from materials (wound, blood) from surgical patients may also be due to the aggressive broad-spectrum antibiotic policy. We should not ignore the local prevalence of ESBL when drug delivery algorithms are constructed.

Based on previous experience concerning research in Polish hospitals, we know that clonal spread more often affects *Klebsiella pneumoniae* strains than *E. coli*; hence, we have now checked whether the high ESBL share among this species is not associated with clonal outbreak [21, 23]. However, the diversity of *Klebsiella* strains in PFGE was relatively high—only 4 strains belonged to one clone. This fact was different from our previous experience; probably, the cases of infection in this study were not associated with the horizontal transfer.

The high prevalence of ESBLs among *Enterobacterales* in hospital wards cannot be unambiguously associated with a high percentage of ESBLs in the general population, and studies conducted by Ny et al. among volunteers in six European countries, including Poland, showed a prevalence of 8% in the case of *E. coli* ESBL and 0% in *K. pneumoniae* ESBL among polish carriers in Silesian Voivodeship [24].

In the light of the data presented, rapid implementation of targeted treatment, especially based on sensitive and quick diagnostic tests, is of particular importance for successful infection treatment. In addition to species identification, more and more commercially available kits for identifying bacteria from blood by molecular methods are able to detect the most popular resistance mechanisms, e.g., KPC, NDM, *bla*_OXA-48_, and others. In Poland, there are currently no clear recommendations on how to effectively implement this type of procedure, and for many hospitals and laboratories, the costs of modern tests may also be a barrier [25]. Our study has a few limitations. It is generally assumed that *E. coli* and *K. pneumoniae* BSI behave similarly, since such a combination was commonly adopted in the literature. Clinical data regarding the hospitalization period were available, and we could analyze only the in-hospital outcome. Previous history of hospitalization was unknown, and we did not know if the patient had previously used antibiotics or had an underlying disease.

## Figures and Tables

**Table 1:** Prevalence of *Enterobacterales* bacilli in studied surgical wards amount 997 patients with BSI.

Studies isolates	*Enterobacterales*			All *n* **=** 121
	*E. coli n* = 74	*Klebsiella* spp. *n* = 33	Others *n* = 14	
Prevalence rate^∗^ (%)	7.4	3.3	1.4	12.1

Surgery wards				
General ward	51 (68.9%)	27 (81.8%)	9 (64.3%)	87 (71.9)
Urology ward	19 (25.7%)	5 (15.1%)	3 (21.4%)	27 (22.3)
Gynecology and obstetrics ward	2 (2.7%)	1 (3.0%)	1 (7.1%)	4 (3.3)
Vascular ward	2 (2.7%)	0 (0.0%)	1 (7.1%)	3 (2.5)

Age range				
≤59	22 (29.7%)	6 (18.1%)	5 (35.7%)	33 (27.3)
60–69	22 (30%)	16 (48%)	3 (21%)	41 (33.9)
70≤	30 (40.5%)	11 (33.3%)	6 (42.8%)	47 (38.8)

Total	74 (61.1%)	33 (27.2%)	14 (11.5%)	121 (100)

^*∗*^Calculated by dividing the number of strains by the number of laboratory-confirmed bloodstream infections and multiplying by 100.

**Table 2:** Drug resistance in *Enterobacterales* bacilli in studied surgical wards.

			*Enterobacterales n* = 121 (100%)		
Nr category	Antimicrobial category	Antimicrobial agent	*E. coli n* = 74 (61.1%)	*Klebsiella* spp. *n* = 33 **(**27.2%)	Others *n* = 14 (11.5%**)**
1	Penicillins and penicillins with inhibitors	Ampicillin	73 (98.6%)	33 (100%)	14 (100%)
		Ampicillin-sulbactam	72 (97.2%)	33 (100%)	13 (92.8%)
		Amoxicillin-clavulanic acid	34 (45.9%)	20 (60.6%)	5 (35.7%)
		Piperacillin-tazobactam	10 (13.5%)	11 (33.3%)	3 (21.4%)

2	Cephalosporins	Cefuroxime IV	29 (39.1%)	15 (45.4%)	7 (50.0%)
		Ceftazidime	29 (39.1%)	14 (42.4%)	5 (35.7%)
		Cefotaxime	28 (37.1%)	13 (39.3%)	5 (35.7%)
		Cefepime	22 (29.7%)	16 (48.4%)	5 (35.7%)
		Cefoperazone-sulbactam	6 (8.1%)	6 (18.1%)	0

3	Carbapenems	Ertapenem	0	1 (3.0%)	0
		Imipenem	1 (1.3%)	0	0
		Meropenem	0	0	0

4	Fluoroquinolones	Ciprofloxacin	36 (48.6%)	24 (72.7%)	8 (57.1%)

5	Aminoglycosides	Gentamicin	19 (25.6%)	7 (21.2%)	5 (35.7%)
		Amikacin	11 (14.8%)	13 (39.3%)	2 (14.2%)
		Tobramycin	24 (32.4%)	17 (51.5%)	5 (35.7%)

6	Others	Trimethoprim-sulfamethoxazole	37 (50.0%)	21 (63.6%)	9 (64.2%)

Nonsusceptible to antimicrobial categories∗		0 categories (fully susceptible)	0	0	0
		1 category	25 (33.7%)	5 (15.1%)	2 (14.2%)
		2 categories	15 (20.2%)	6 (18.1%)	3 (21.4%)
		3 categories	4 (5.4%)	4 (12.1%)	4 (28.5%)
		4 categories	11 (14.8%)	4 (12.1%)	1 (7.1%)
		5 categories	18 (24.3%)	14 (42.4%)	4 (28.5%)
		6 categories	1 (1.36%)	0	0

^*∗*^Strains were divided into six categories based on their resistance to a specific number of antimicrobial agent categories (penicillin and penicillins with inhibitors, cephalosporins, carbapenems, fluoroquinolones, aminoglycosides, and trimethoprim-sulfamethoxazole); fully susceptibility to all antimicrobial agents from all categories means “0”; “6” means resistance to at least one antimicrobial agent from each of the 6 categories.

**Table 3:** Prevalence of ESBL strains among *Enterobacterales* bacilli in studied surgical wards.

Studied isolates	*E. coli n* = 74 (100%)	*Klebsiella* spp. *n* = 33 (100%)	Others *n* = 14 (100%)	All isolates *n* = 121 (100%)
ESBL (yes, %)	19 (25.7%)	22 (66.7%)	4 (28.6%)	45 (37.2%)
General ward	15 (20.3%)	17 (51.5%)	3 (21.4%)	35 (28.9%)
Urology ward	3 (4.0%)	4 (12.1%)	1 (7.1%)	8 (6.6%)
Gynecology and obstetrics ward	1 (1.4%)	1 (3.0%)	0 (0%)	2 (1.6%)
Vascular ward	0 (0%)	0 (0%)	0 (0%)	0 (0%)

## Data Availability

The data used to support the findings of this study are available from the Department of Microbiology of the Jagiellonian University Medical College upon request (Agnieszka Chmielarczyk, agnieszka.chmielarczyk@uj.edu.pl and Jadwiga Wojkowska-Mach, jadwiga.wojkowska-mach@uj.edu.pl). Data on drug resistance of strains are also available in the KORLAB laboratory (Monika Pomorska-Wesołowska monikapw@op.pl).
